# Evolutionary aspects of non-cell-autonomous regulation in vascular plants: structural background and models to study

**DOI:** 10.3389/fpls.2014.00031

**Published:** 2014-02-11

**Authors:** Anastasiia I. Evkaikina, Marina A. Romanova, Olga V. Voitsekhovskaja

**Affiliations:** ^1^Laboratory of Plant Ecological Physiology, Komarov Botanical Institute, Russian Academy of SciencesSaint Petersburg, Russia; ^2^Department of Botany, Saint Petersburg State UniversitySaint Petersburg, Russia

**Keywords:** Lycopodiophyta, primary plasmodesmata, secondary plasmodesmata, cell boundaries, shoot apical meristem, KNOX transcription factors

## Abstract

Plasmodesmata (PD) serve for the exchange of information in form of miRNA, proteins, and mRNA between adjacent cells in the course of plant development. This fundamental role of PD is well established in angiosperms but has not yet been traced back to the evolutionary ancient plant taxa where functional studies lag behind studies of PD structure and ontogenetic origin. There is convincing evidence that the ability to form secondary (*post-cytokinesis*) PD, which can connect any adjacent cells, contrary to primary PD which form during cytokinesis and link only cells of the same lineage, appeared in the evolution of higher plants at least twice: in seed plants and in some representatives of the Lycopodiophyta. The (in)ability to form secondary PD is manifested in the symplasmic organization of the shoot apical meristem (SAM) which in most taxa of seedless vascular plants differs dramatically from that in seed plants. Lycopodiophyta appear to be suitable models to analyze the transport of developmental regulators via PD in SAMs with symplasmic organization both different from, as well as analogous to, that in angiosperms, and to understand the evolutionary aspects of the role of this transport in the morphogenesis of vascular plant taxa.

## THE APPEARANCE OF PD IN EVOLUTION

Plasmodesmata (PD) are tiny channels of several tenths to hundreds of nanometers in diameter which connect adjacent cells providing continuity of their cytoplasm and plasma membranes, and in most cases also continuity of their endoplasmic compartments and endoplasmic reticulum (ER) membranes due to the presence of the desmotubules. In multicellular organisms, PD provide a convenient route for cell-to-cell communication among immobile cells surrounded by rigid cell walls. In fact, PD occur in land plants, in multicellular algae and phototrophic protists (but astonishingly not in all groups; reviewed by Raven, 2005), as well as in a few species of multicellular fungi (reviewed by Lucas et al., 1993). An excellent review by Raven (2005) indicates that PD evolved independently in brown algae, in characean algae, and up to five times in green algae. Although truly multicellular forms can be found also among red algae, haptophytes and dinoflagellates, they do not form PD.

Depending on the stage of the cell cycle during which they appear, PD are classified as primary, or cytokinetic, and secondary, which form *post-cytokinesis*. Primary PD can be secondarily modified later by addition of new branches, or serve as templates for secondary PD formation (Ehlers and Kollmann, 2001; Faulkner et al., 2008). The mechanism of primary PD formation strictly correlates with the mechanism of cell division: primary PD occur between cells which divide via the formation of a cell plate and not via furrowing (Stewart et al., 1973), with the possible exception of a few brown algae (Katsaros et al., 2009). In characean algae and embryophytes, after separation of the nuclei, the microtubules of the mitotic spindle remain perpendicular to the plane of cell division where the nascent phragmoplast forms. Cisterns of the ER are laid along these microtubules and serve as templates for the primary PD, becoming the desmotubules later on. Interestingly, in the PD-forming fungus *Rhizopus sexualis* cell divisions occur similarly to those of land plants, i.e., via the formation of a phragmoplast, with the difference that the cell plate forms centripetally and not centrifugally; PD in this fungus contain desmotubules (Hawker and Gooday, 1967). In green algae, the cell plate formation is accompanied (after the mitotic spindle has disappeared) by the formation of a system of microtubules oriented parallel to the plane of cell division, called phycoplast. Although there is no obvious mechanism for insertion of desmotubules in PD, in a few green algae, e.g., *Ulothrix* and *Stigeoclonium* (Floyd et al., 1971) PD were shown to contain desmotubules. In brown algae, cell plate formation involves neither phycoplast nor phragmoplast; an elegant study on *Dictyota dichotoma* has shown that the pre-PD, probably synthesized in the cytoplasm as whole complex structures, are introduced into membranous sacs positioned at the place of the future cell plate (Terauchi et al., 2012). The PD in brown algae typically do not contain desmotubules (Katsaros et al., 2009).

The occurrence of secondary PD in the absence of primary PD has been reported for several algae. One example is *Chara corallina* where simple PD lacking desmotubules are formed as holes appearing in already existing cell walls (Franceschi et al., 1994). Interestingly, Brecknock et al. (2011) recently reported the presence of desmotubules in PD of the same species. In *Chara zeylanica*, the formation of desmotubules-containing primary PD resembling those in embryophytes has been observed while no secondary PD have been found (Cook et al., 1997). Thus, there are characean algae with only primary PD, as well as characean algae with only secondary PD; to our knowledge, no characean algae capable of forming both primary and secondary PD, similarly to seed plants, have been found thus far.

An interesting hypothesis on the appearance of PD in plant evolution has been proposed by Niklas (2000). He argued that “some of the features characterizing the multicellular plant body, such as cellular differentiation, PD-like structures, control mechanisms for the orientation of cell cleavage, and cellular differentiation, are evident among extant and presumably very ancient cyanobacteria.” Thus, such traits like cell wall and PD might have evolved as parts of the phenomenon of multicellularity which could have been transmitted from cyanobacterial endosymbionts to the host nucleus via lateral transfer of genes “during or shortly after primary endosymbiotic events in the very distant past” (Niklas, 2000). Other authors argued that this seems rather unlikely, as the intercellular connections in cyanobacteria are more similar to gap junctions than to PD (Raven, 2005). However, the newest findings indicating that the process of PD formation in plant embryos depends to some extent on plastid signals (Burch-Smith and Zambryski, 2010; Burch-Smith et al., 2011), support Niklas’ hypothesis.

Several first class transmission electron microscopy (TEM) studies on PD in algae revealed a range of different internal structures which obviously reflect different phylogenetic and ontogenetic origin of these PD (Fraser and Gunning, 1969; Franceschi et al., 1994; Cook et al., 1997; Brecknock et al., 2011; Terauchi et al., 2012 and others). It can be expected that the composition of the cell wall, as well as the chemistry of the lipids in plasma membrane and in the ER for the desmotubule-containing PD, will have an impact on the mechanisms of PD functioning, e.g., regulation of the size exclusion limit (SEL), which might be very different in PD of different origin. So far, no data on the role of PD in the regulation of development are available for algae and fungi.

## WHICH FACTORS MAY INFLUENCE PD FORMATION AND FUNCTIONS IN SEEDLESS VASCULAR PLANTS AS COMPARED TO SEED PLANTS?

**FIGURE 1:**
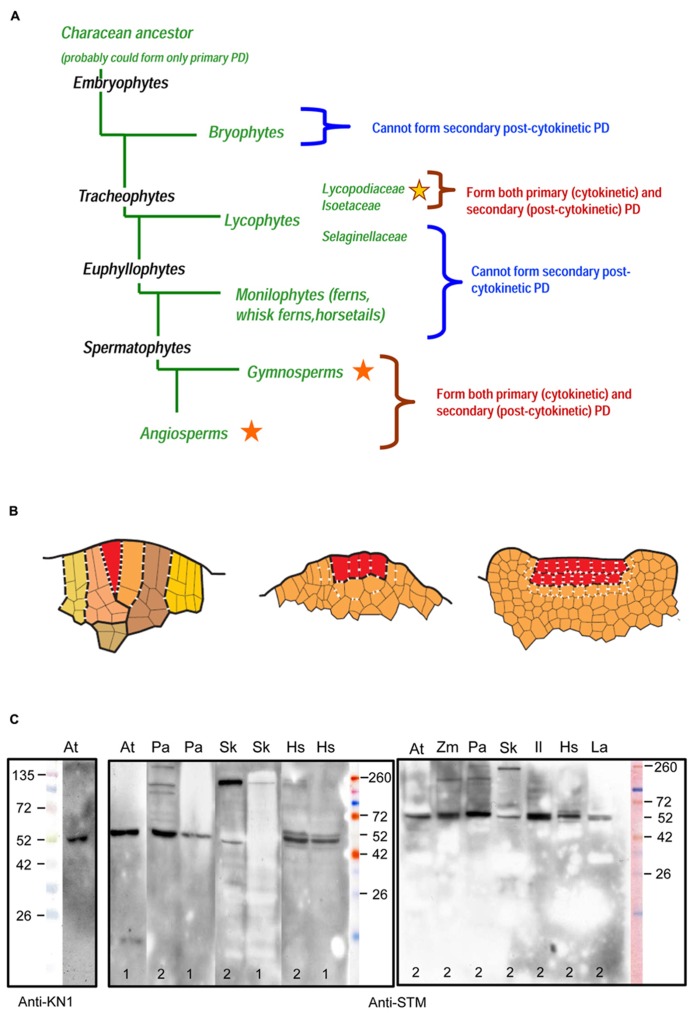
**(A)** Occurrence of primary and secondary plasmodesmata (PD) in sporophytes throughout the main groups of embryophytes (Kenrick and Crane, 1997; Judd et al., 2002). Stars indicate the presence of secondary PD; different colors of stars stand for independent evolutionary origins. **(B)** Schematized sections of three types of the SAM symplasmic structure in sporophytes of land plants. Line drawings are based on original unpublished data of the authors for *Selaginella kraussiana ***(A)**, *Huperzia selago ***(B)** and *Syringa vulgaris ***(C)**. Different colors in **(A)** mark successive segments of the single apical cell (merophytes) and their derivatives. Single and plural apical initials in **(A) **and **(B)** are marked with red color. In **(C)** red color marks cell layers of the tunica. Primary PD are symbolized by dashes, secondary PD by crosses. The numbers of dashes and crosses per cell wall do not reflect actual PD density but roughly (in limits of one order of magnitude) correspond to the original unpublished data of the authors for *Selaginella kraussiana* and *Huperzia selago*, and to the data of Imaichi and Hiratsuka (2007) for Lycopodiaceae, Selaginellaceae, and Isoetaceae species. **(A)** Lineage-specific networks of primary PD in ferns and lycophytes from the Selaginellaceae family. **(B)** Interface-specific network of primary and secondary PD in lycophytes from the Lycopodiaceae and Isoetaceae families; primary and secondary PD seem to be distributed randomly. **(C)** Interface-specific network of primary and secondary PD in angiosperms; basal periclinal walls of tunica cells form long-persisting cellular boundaries equipped exclusively with specialized (secondary) PD. **(C)** Specificity of an antibody raised against a peptide region of the *Arabidopsis thaliana* shoot meristemless (STM) protein to KNOX homologs from a range of embryophyte taxa (http://www.agrisera.com/en/artiklar/stm-homeobox-protein-shoot-meristemless.html). Immunoblots of 10 μg of total protein extract per slot are shown for *A. thaliana *2-week-old seedlings (At), *Picea abies* shoot apices (Pa), *Huperzia selago* shoot apices (Hs), *Selaginella kraussiana* shoot apices (Sk), *Lycopodium annotinum* shoot apices (La), *Isoetes lacustris* shoot apices (Il), and *Zea mays* shoot apices (Zm). Proteins were extracted either according to Conlon and Salter (2007; 1) or Carpentier et al. (2005; 2). Details of protein separation, blotting and development of the blots are published on the Agrisera website (see above). An anti-KN1 antibody raised against *Z. mays* KNOTTED1 protein (Smith et al., 1992) was used with protein extracts from Arabidopsis seedlings. Primary antibodies were diluted 1:5000. In *Arabidopsis*, both antibodies recognize a single band of about 52 kDa which is running higher than the predicted molecular weight (MW) of STM protein (42.7 kDa). Similar results were obtained with anti-KN1 antibody on immunoblots with *Z. mays* protein extracts: while the predicted MW for ZmKNOTTED1 is 39.0 kDa, single bands recognized by the antibody showed an apparent MW of 42 kDa (Smith et al., 1992), 50 kDa (Harrison et al., 2005), or of 47 kDa (Nowak et al., 2011). Predicted molecular masses of *Arabidopsis* STM and its known homologs: *A. thaliana *STM (AEE33958.1), 42.7 kDa; *Picea abies *homeobox protein (AAC84001.1), 48.6 kDa; *Selaginella kraussiana* SkKNOX1 (AAW62517.1), 51.4 kDa. For the other species on these blots, the sequences of the homologs are unknown.

Embryophytes have evolved from a characean ancestor (**Figure 1A**) but there are significant differences in membrane lipids and cell wall composition, as well as in the hormonal regulatory networks, between charophytes at the base and angiosperms at the top of land plant evolution, which can potentially impact the structure and function of PD in different taxa of embryophytes. PD of angiosperms are the best studied among land plants in terms of substructure, formation, and function. Their important properties include the presence of two closely juxtaposed membranes of different origin, the plasma membrane and the desmotubule, which probably contain special lipid domains, e.g., sphingolipid-enriched lipid rafts (Tilsner et al., 2011); the enclosure into a cell wall sheath which consists of the non-esterified pectin homogalacturonan and of callose and is devoid of cellulose and hemicelluloses (Heinlein and Epel, 2004); the ability to facilitate the intercellular spread of viral RNA, proteins and whole virions, as well as to transport non-cell-autonomous proteins and regulatory RNA, and the ability to reversibly change their diameter (“to gate”), possibly via regulation of callose synthesis and degradation (Lucas et al., 1993, 1995; Epel, 2009).

In seedless plants, the knowledge on cell wall composition, lipidomics, and hormonal regulatory networks is limited compared to angiosperms. However, genome sequencing in the moss *Physcomitrella patens* and the spike moss *Selaginella moellendorffii* has recently provided a solid basis for future analyses of evolution and diversity of these and other aspects in land plants. Several points might be potentially important for the study of PD function in non-angiosperms, as concluded from the data for angiosperms. One is the composition of the cell wall which sometimes influences the structure of PD, as found, e.g., in sugarcane where the presence of suberin lamellae resulted in the constriction of the desmotubules in PD (Robinson-Beers and Evert, 1991). Also, as mentioned above, in angiosperms PD were shown to be surrounded by a cell wall sheath of specific composition devoid of cellulose and enriched in low-esterified homogalacturonan: this was demonstrated by Roy et al. (1997) for PD in ripe apple and by Sutherland et al. (1999) for PD in kiwi fruit (reviewed in Heinlein and Epel, 2004). The reason for this is not known; however, it can be speculated that the presence of these specific cell wall constituents is important for PD biogenesis and/or function. No data on cell wall domains around PD are available for non-angiosperms thus far.

Cell wall composition in land plants shows significant variations. Characean algae possess only primary cell walls which do not contain lignin or rhamnogalacturonan II, while mannose-containing polymers are present in high amounts. In bryophytes, cell wall composition generally resembles that in charophytes, while the cell walls of lycophytes and monilophytes contain typical “angiosperm” hemicelluloses and pectins, and can be secondarily lignified. Seedless plants contain hydroxyphenyl and guaiacyl lignins while *Selaginella moellendorffii* possesses an angiosperm-type syringyl lignin, an example of convergent evolution (Weng et al., 2008; Weng and Chapple, 2010). Homogalacturonan, an important component of the pectin enclosing PD in angiosperms, in *Selaginella moellendorffii* shows a recalcitrance to the typical extraction methods which might indicate the presence of specific modifications (Harholt et al., 2012). A special kind of mixed beta-1-3- and beta-1-4-linked glucans (MLG) typical of Poaceae has been found in *Selaginella moellendorffii* which is another example of convergent evolution, since in *Selaginella moellendorffii*, MLG is synthesized by enzymes which are non-homologous to those in angiosperms (Harholt et al., 2012). Altogether, the assortment of hemicellulose and pectins in cell walls of seedless plants is not the same as in seed plants. Contrarily, callose, which is, perhaps, the most important regulator of PD diameter in angiosperms, has been found in characean algae and throughout all embryophyte taxa (Fangel et al., 2012). Another potentially important point for PD functions is the fact that seedless plants have an evolutionary ancient complement of sphingolipids (which, however, has been also detected in Poales as a further example of convergent evolution; Nurul Islam et al., 2012), as sphingolipids represent an important component of lipid rafts in plants (Mongrand et al., 2010), structures which recently were speculated to be involved in PD function (Tilsner et al., 2011).

Another peculiarity which might have been expected to have some impact on PD formation is monoplastidy of some cells in bryophytes and in the lycophyte genera *Selaginella* and *Isoetes*, including cells in their apical meristems (Brown and Lemmon, 1984, 1990). Monoplastidy introduces some important changes into the mechanism of mitosis: to ensure that each sister cell receives a plastid, an unusual plastid-based mitotic spindle is formed during monoplastidic divisions. This changes the geometry of the microtubules in the mitotic spindle as compared to the situation in dividing polyplastidic cells, in that microtubules are not uniformly distributed over the region of cell plate formation and lay perpendicular to it, but are bound on two foci at the plastid. Thus, it might be expected that the pattern of primary PD laid along these microtubules also changes accordingly. However, so far the formation of phragmoplasts and of primary PD has not been reported to be influenced by the involvement of plastids in cell division in these plants. Generally, the structure of PD in seedless plants was found to be similar to that in seed plants; there is, to our knowledge, only one exception, namely, the unique structure of expanded PD in angle and apical meristems of *Selaginella willdenovii* (Wochok and Clayton, 1976).

A prominent feature of PD in angiosperms is their participation in the cell-to-cell spread of viruses. Interestingly, there are very few, altogether seven, reports on virus infection and spread in seedless vascular plants (Valverde and Sabanadzovic, 2009 and references therein; Scheets et al., 2011 and references therein), including a report on a new previously unknown RNA virus found in the fern *Cyrtomium falcatum* which was assigned to a new taxon named Pteridovirus (Valverde and Sabanadzovic, 2009). Infected plants of *Cyrtomium falcatum* showed visible symptoms indicative of spread of this virus via PD; the infection was shown to be transmitted from infected to healthy ferns by grafting and from spore to spore, while attempts to transmit the virus to several angiosperm species by injury failed (Valverde and Sabanadzovic, 2009). The extremely rare occurrence of viral diseases in seedless plants, as well as the unusual properties of this virus which is probably limited to fern hosts only, might indicate important differences in the organization of PD and the regulation of cell-to-cell transport of macromolecules in seedless plants. Another indication comes from the analysis of genome in *Selaginella moellendorffii* (Banks et al., 2011). First, the trans-acting small interfering RNA (tasiRNA) pathway is lacking in this species. tasiRNA represent silencing signals known to act non-cell-autonomously and spread locally over about a 10–15-cell-distance in angiosperms (reviewed by Hyun et al., 2011). Second, the proportion of the small RNA of 24 nt in length, which are known to spread systemically over long distances, is very low in *Selaginella moellendorffii*. Altogether, these data might indicate that in ancient taxa of vascular plants, the ability of PD to mediate the transport of viral and endogenous RNAs is much lower than in angiosperms.

Information on the regulation of PD biogenesis and of transport through PD is yet scarce. Transport through PD is regulated by turgor pressure differentials in tobacco leaf trichomes (Oparka and Prior, 1992) and in *Chara corallina* (Ding and Tazawa, 1989), and has been proposed to be regulated in a similar manner in PD between mesophyll and phloem companion cells (Voitsekhovskaja et al., 2006). PD SEL and the spatial distribution of complex PD in *Arabidopsis* leaves were recently suggested to be the subject of osmotic regulation (Fitzgibbon et al., 2013). Although there are indications for transport of hormones through PD, few data on the hormonal regulation of PD function are available for angiosperms. Gibberellins were shown to influence callose turnover at PD through up-regulation of glucan hydrolase family genes, leading to the reopening of signal conduits and to the release of dormancy after chilling in *Populus* (Rinne et al., 2011). Salicylic acid (SA) has been found to accelerate the formation of complex PD up to threefold in *Arabidopsis* leaves (Fitzgibbon et al., 2013). As complex PD are characterized by a reduced SEL, their enhanced formation in response to SA might be a part of the response to pathogen stress that reduces the non-selective movement of pathogen-induced macromolecules or viruses between host cells. Another aspect of the role of SA in the innate immune response involves the induction of callose accumulation around PD via up-regulation of specific callose synthases (Wang et al., 2013).

Cytokinins, auxins, abscisic acid (ABA), brassinosteroids, and ethylene are ancient plant hormones already present in algae, but the responses to these hormonal signals evolved to a different extent in various embryophyte taxa (Paponov et al., 2009; Pils and Heyl, 2009; Prigge and Bezanilla, 2010; Kutschera and Wang, 2012; McAdam and Brodribb, 2012; Yasumura et al., 2012). Gibberellins and jasmonates evolved later. Components of the jasmonate biosynthesis are present in *P. patens* but jasmonate is not synthesized (Stumpe et al., 2010). No data on the jasmonate biosynthesis pathway in lycophytes are available, but Li et al. (2012) observed the production of volatile terpenes (induced by jasmonate in seed plants) in *Selaginella moellendorffii* in response to fungal elicitors, similar to seed plants, although the enzymes of the pathway are partially encoded by unique genes not homologous to their angiosperm equivalents. Key components of gibberellic acid signaling are present in the genome of *Selaginella moellendorffii* but not of *P. patens* (Schwechheimer and Willige, 2009). In angiosperms, cytokinins were reported to promote secondary PD formation (Ormenese et al., 2000, 2006), while the ratio of ABA to cytokinins was shown to influence the ultrastructure of PD (Botha and Cross, 2000). As only primary PD have been found in bryophytes, in the lycophyte family Selaginellacae, and in ferns, while secondary PD in the Lycopodiaceae and Isoetaceae families of the lycophytes evolved independently from those in seed plants (see below), it would be interesting to investigate the effects of cytokinin on PD formation and function in these groups.

A recently discovered fascinating facet is the regulation of PD formation and transport via PD in angiosperms by reactive oxygen species and plastid signals (Benitez-Alfonso et al., 2009; Burch-Smith et al., 2011; Burch-Smith and Zambryski, 2012; Stonebloom et al., 2012). One example of this is the regulation of PD in *Arabidopsis* roots by different levels of hydrogen peroxide (Rutschow et al., 2011). This has never been investigated in ancient plant taxa.

## THE EVOLUTION OF SECONDARY PD AND SHOOT APICAL MERISTEM ORGANIZATION IN LAND PLANTS

Although both primary and secondary PD occur in angiosperms, a special role in transport of developmental regulators, signals, and viruses has been assigned to secondary PD (Ding et al., 1992, 1993; Ding and Lucas, 1996). Ding et al. (1993) hypothesized that secondary PD differ from primary PD in being able to transport developmental signals. Ding et al. (1992) found that PD between mesophyll cells of the leaf, which are primary PD by origin with secondary modifications added later on, differ from the originally secondary PD at the interface between phloem companion cells and bundle sheath cells in that tobacco mosaic virus movement protein (TMV-MP) gates the former but not the latter PD (Ding et al., 1992; but see Crawford and Zambryski, 2001). Non-cell-autonomous transcription factors are synthesized in one cell and travel through PD to act in another cell. Kim et al. (2003) studied in transgenic *Arabidopsis* the traffic of fusions of GFP with KN1, the non-cell-autonomous transcription factor KNOTTED1 from *Zea mays* (Hake and Freeling, 1986) which functions in the shoot apical meristem (SAM). They found that GFP-KN1 is able to traffic from the L1 into the L2 meristematic layer in the SAM but not further, and in mature leaves, GFP-KN1 traffics from mesophyll cells into the epidermis (produced by the L2 and L1 SAM layers, respectively) but not inversely. Interestingly, in maize SAMs, KNOTTED1 traffics from the L2 into the L1. Since PD between L1 and L2 are secondary in origin, these data seem to suggest that secondary PD in angiosperms are specifically equipped to enable the transport of non-cell-autonomous developmental regulators like KNOTTED1. On the other hand, the observations that a number of transcription factors, e.g., CPC and SHR (Kurata et al., 2005; Rim et al., 2011), can move also between cells of the same lineage connected via primary PD (e.g., trichoblasts and atrichoblasts or endodermis and cortex, respectively), seem to contradict this suggestion.

Studies on fern gametophytes and on the apical meristems of fern sporophyte roots and shoots have shown that here, all PD arise exclusively during cytokinesis, i.e., are primary (Gunning, 1978; Gunning et al., 1978; Tilney et al., 1990; Imaichi and Hiratsuka, 2007). Strikingly, the (in)ability to form secondary PD is reflected by the symplasmic organization of the SAM. In ferns, SAMs possess a single initial, also named the apical cell, which produces merophytes in a highly regular fashion. All cell walls observed within the SAM are formed recently, as the merophytes are rapidly displaced by newly formed ones. Ultrastructural and developmental studies in ferns revealed several mechanisms regulating PD numbers between the cells. First, “an abrupt increase in plasmodesmatal number occurs in the cell wall that is destined to construct one side of the future apical cell before that apical cell appears” (Tilney et al., 1990). Second, in the course of merophyte cell divisions, the number of PD in the newly formed cell walls is determined so as to match the PD density of the already existing cell walls, while taking into “consideration” the future expansion of this newly formed cell wall (Gunning, 1978). Third, before the cessation of growth, PD numbers decrease in the walls of the last subapical cells formed from the senescent apical cell (Gunning, 1978). Thus, PD amount and density in cell walls of mature cells depend on the initial amount of PD laid by the apical cell during its division, on the extent of cell wall expansion, on the total number of cell divisions and on eventual loss by occlusion. This results in a PD density gradient starting from the apical cell, where the PD densities are among the highest reported thus far (Cooke et al., 1996). Moreover, the potential to grow indefinitely might be limited by the numbers of PD between the cells in ferns (Gunning, 1978). In seed plants, SAMs possess multiple initials organized in several cell layers. The periclinal cell walls between the layers persist within the meristem during its life, and sometimes can even be traced back to the protoderm of the embryo (Cooke et al., 1996). As the cells in SAMs of seed plants can form secondary PD, there are no visible gradients of PD density. On the basis of the analyses of PD densities and distribution patterns in fern and seed plant root and SAMs, Cooke et al. (1996) proposed two main types of PD network organization in embryophytes: the lineage-specific network of primary PD which connect cells of the same lineages and is found in ferns, and the interface-specific network of primary and secondary PD which can connect any adjacent cells and is typical for seed plants. The type of PD network strongly correlates with the organization of the SAM in that SAMs with single initials have the lineage-specific network of primary PD and those with plural initials have the interface specific network of primary and secondary PD, respectively (Cooke et al., 1996).

It is generally accepted that embryophytes evolved from a characean ancestor forming exclusively primary PD (Cook et al., 1998). At what time did the ability to form secondary PD appear in the evolution? In Lycopodiophyta, a sister group to other vascular plants, SAMs with single initials and PD density gradients are found in representatives of the Selaginellaceae family, while SAMs with multiple initials and evenly distributed primary and secondary PD are found in representatives of the Isoetaceae and the Lycopodiaceae families (Imaichi and Hiratsuka, 2007). This raises the question whether the ability to form secondary PD appeared independently in seed plants and some Lycopodiophyta, or was lost in ferns. PD formation and distribution in bryophytes follow the fern pattern both in the gametophyte and the sporophyte SAMs, which indicates an independent origin of secondary PD in Lycopodiophyta (Ligrone and Duckett, 1998; Mansouri, 2012; **Figure 1A**). However, observations on *Sphagnum palustre* leaflet development showed that PD density did not decrease in the course of equal cell divisions, and even increased after the first unequal division (Schnepf and Sych, 1983). This might suggest that the mechanism for secondary PD development evolved in bryophytes, although as a homoplasy, as it occurs only in the gametophyte generation.

Formation of secondary PD is considered necessary for successful graft unions. Interestingly, there is only one record of grafting lycophytes, namely from Daniel (1901) for *Selaginella arborea *(now *Selaginella willdenovii*), and to our knowledge grafting of lycophytes has never been reported since. However, grafting is indeed possible in ferns (e.g., Valverde and Sabanadzovic, 2009), indicating that, in spite of the absence of secondary PD in their tissues, ferns, but probably not lycophytes, can form secondary PD in special cases.

These data indicate that the cell boundaries in SAMs of “fern” and of “seed plant” types, respectively, are specified by different mechanisms, raising the question whether transport of developmental regulators via PD occurs similarly in SAMs of seed plants, in the “fern” type SAMs and in the “seed plant” type SAMs of Lycopodiaceae containing secondary PD of independent evolutionary origin. In SAMs of the fern type, the informational exchange via PD between cells and merophytes seems to be only possible within cell lineages. The fact that cell walls are rapidly displaced from the SAM might make it impossible to establish a long-persisting boundary equipped with specialized PD between meristematic domains, like those between the L1, L2, and L3 cell layers in angiosperms SAMs. The absence of secondary PD raises the question whether primary PD are able to exert efficient control over selective traffic of developmental signals in “fern” type SAMs. The SAMs of “seed plant” type in Lycopodiophyta are organized similarly to those in angiosperms in that they contain both primary and secondary PD, but the independent evolutionary origin of secondary PD makes it probable that the transport via these PD is regulated by different mechanisms. At the same time, contrarily to the SAMs of seed plants, the SAMs of Lycopodiaceae seem to contain no long-persisting boundaries between meristematic domains. In **Figure 1B**, we show schematized depictions of the three types of the SAMs organization.

## LYCOPHYTES AS THE MODELS TO STUDY EVOLUTION OF NON-CELL-AUTONOMOUS TRANSPORT OF DEVELOPMENTAL REGULATORS IN VASCULAR PLANTS

Lycophyte species belonging to the Selaginellaceae and Lycopodiaceae/Isoetaceae, respectively, seem to be the most interesting models for studies of non-cell-autonomous transport in ancient plant taxa, in our opinion, because they would allow to analyze the trafficking of developmental regulators in SAMs with two most contrasting symplasmic organization within the same taxon Lycopodiophyta. Another strong argument for the studies of Lycopodiophyta is the current availability of the only genome from a seedless vascular plant, namely that of *Selaginella moellendorffii*. Nevertheless, in the future, studies of PD function in the development in all taxa of land plants will be necessary to complete the evolutionary view of the establishment of this phenomenon. In angiosperms, the best studied example of non-cell-autonomous regulators in SAMs are KNOX transcription factors (Jackson, 2005). Recently, Chen et al. (2013) could show on the basis of trichome rescue assays the non-cell autonomous mode of action of KNOX proteins from the moss *P. patens*, but not from the unicellular alga *Chlamydomonas*, and concluded that the ability to efficiently traffic through PD may have been acquired early in the evolution of land plants for KNOX homeodomain proteins. In bryophytes, KNOX genes play a role in sporophyte development (Sakakibara et al., 2008). The functions of KNOX genes are conserved among lycophytes, ferns and angiosperms (Harrison et al., 2005). Comparison of the cellular distribution patterns of the gene transcripts and proteins, respectively, can give an insight into the non-cell-autonomy of the KNOX protein homologs in seedless plants (Jackson, 2002). KNOX gene homologs have been cloned in some seedless vascular plant species, and the localization of their transcripts and encoded proteins have been performed in several cases (**Table 1**). Unfortunately, none of the studies compared patterns of transcript and protein localization in the same species at the cellular and tissue level, so at present, no conclusions can be drawn about the non-cell-autonomy of the KNOX proteins in seedless plants. For such studies, obtaining high-resolution cytological pictures, which is rather challenging for SAMs with a single initial, may become a critical point. Another important aspect is the availability of the antibodies able to specifically recognize KNOX homologs in SAMs of seedless plants. We analyzed the specificity of several antibodies raised against KN1 protein from *Z. mays* (Smith et al., 1992) and against shoot meristemless (STM) from *Arabidopsis* (Agrisera, Sweden) on Western blots with total protein extracts from representatives of a range of embryophyte taxa using two different extraction methods (**Figure 1C**). The antibodies recognized, depending on the species and the extraction method, one to several KNOX protein homologs, showing that they can be used for immunohistochemical studies in lycophytes. Transcriptome analyses of shoots of Lycopodiaceae species, along with the available data on *Selaginella moellendorffii KNOX* gene homologs, may provide a tool for analyses of KNOX gene transcript localization in SAMs of lycophytes to be compared with the localization of the proteins. Along with *Selaginella moellendorffii*, *Selaginella kraussiana *is another convenient model for such studies because KNOX genes have been cloned and studied in this species (Harrison et al., 2005). *Huperzia selago* is a representative of the Lycopodiaceae family which is characterized by a highly ordered structure of a “seed plant” type SAM with several rectangular apical initials and vigorously dichotomously branching shoots with relatively big apices that can yield sufficient amounts of RNA for molecular studies (Romanova et al., 2010). This species could provide a Lycopodiaceae model to supplement the studies on Selaginellaceae species.

**Table 1:** Localization on KNOX gene products (mRNA, proteins) in seedless vascular plants.

Species	Product of the *KNOX* gene	Localization of the *KNOX* gene product	Reference
**Polypodiophyta**
*Ceratopteris richardii*		Class 1 *KNOX* genes	Sano et al. (2005)
	*CrKNOX1* mRNA	SAM: single initial, surface initial derivatives; leaf primordia; procambium	
	*CrKNOX2* mRNA	SAM: single initial, surface initial derivatives; leaf primordia; procambium	
		Class 2 *KNOX* genes	
	*CrKNOX3*	No data	
*Osmunda regalis*	KNOX protein homolog	SAM: periferal meristem cells, subsurface meristem cells; leaf primordia; procambium	Harrison et al. (2005)
*Anogramma chaeophylla*	KNOX protein homolog	SAM; leaf primordia; procambium	Bharathan et al. (2002)
**Lycopodiophyta**
*Selaginella kraussiana*		Class 1 *KNOX* genes	Harrison et al. (2005)
	*SkKNOX1* mRNA	SAM: subsurface meristem cells	
	*SkKNOX2* mRNA	Internodal regions of the shoot	
		Class 2 *KNOX* genes	
	*SkKNOX3*	No data	
*Selaginella uncinata*		Class 1 *KNOX* genes	Kawai et al. (2010)
	*SuKNOX1 mRNA*	SAM cells including single initial	
		Class 2 *KNOX* genes	
	*SuKNOX2*	No data	

In conclusion, structural data on SAM organization and -function in seedless vascular plants, the presence of two contrasting types of PD networks in lycophytes, and the availability of the genome sequence of *Selaginella moellendorffii*, make functional studies of PD in lycophytes an interesting option which will help to understand the role of intercellular transport via PD in the morphogenesis of vascular plant taxa with different phylogenetic history. The present, yet scarce and indirect information suggests that the ability of the PD to mediate cell-to-cell transport of macromolecules in ancient taxa of vascular plants might differ from that in angiosperms. The comparative study of cellular patterns of localization of transcripts and protein homologs of the known non-cell-autonomous developmental regulators in the representatives of the Selaginellaceae and Lycopodiaceae/Isoetaceae can provide the first evidence in testing this hypothesis.

## Conflict of Interest Statement

The authors declare that the research was conducted in the absence of any commercial or financial relationships that could be construed as a potential conflict of interest.
